# Plant-CNN-ViT: Plant Classification with Ensemble of Convolutional Neural Networks and Vision Transformer

**DOI:** 10.3390/plants12142642

**Published:** 2023-07-14

**Authors:** Chin Poo Lee, Kian Ming Lim, Yu Xuan Song, Ali Alqahtani

**Affiliations:** 1 Faculty of Information Science and Technology, Multimedia University, Melaka 75450, Malaysia; cplee@mmu.edu.my (C.P.L.);; 2 Department of Computer Science, King Khalid University, Abha 61421, Saudi Arabia; 3 Center for Artificial Intelligence (CAI), King Khalid University, Abha 61421, Saudi Arabia

**Keywords:** plant classification, plant leaf classification, deep learning, convolutional neural network, Vision Transformer

## Abstract

Plant leaf classification involves identifying and categorizing plant species based on leaf characteristics, such as patterns, shapes, textures, and veins. In recent years, research has been conducted to improve the accuracy of plant classification using machine learning techniques. This involves training models on large datasets of plant images and using them to identify different plant species. However, these models are limited by their reliance on large amounts of training data, which can be difficult to obtain for many plant species. To overcome this challenge, this paper proposes a Plant-CNN-ViT ensemble model that combines the strengths of four pre-trained models: Vision Transformer, ResNet-50, DenseNet-201, and Xception. Vision Transformer utilizes self-attention to capture dependencies and focus on important leaf features. ResNet-50 introduces residual connections, aiding in efficient training and hierarchical feature extraction. DenseNet-201 employs dense connections, facilitating information flow and capturing intricate leaf patterns. Xception uses separable convolutions, reducing the computational cost while capturing fine-grained details in leaf images. The proposed Plant-CNN-ViT was evaluated on four plant leaf datasets and achieved remarkable accuracy of 100.00%, 100.00%, 100.00%, and 99.83% on the Flavia dataset, Folio Leaf dataset, Swedish Leaf dataset, and MalayaKew Leaf dataset, respectively.

## 1. Introduction

Plant leaf classification refers to the process of identifying and categorizing plant species based on their leaf characteristics. It plays a significant role in various fields, including botany, agriculture, and ecological research. Existing works in plant leaf classification have utilized handcrafted features combined with traditional machine learning [1,2,3,4,5,6,7] or deep learning models [8,9,10,11,12,13,14,15,16,17] for feature extraction and classification. However, these approaches have certain limitations that reduce their effectiveness in accurately classifying plant leaves.

One limitation is associated with handcrafted features used in conjunction with traditional machine learning approaches. These features are manually designed based on domain knowledge and may not fully capture the diverse and complex variations present in plant leaves. They may lack the ability to represent the intricate details and subtle differences among leaf species accurately. Furthermore, traditional machine learning algorithms may struggle to learn complex patterns and hierarchical representations from these handcrafted features, resulting in limited classification accuracy. On the other hand, deep learning models have demonstrated great potential in various image classification tasks. However, deep learning models trained from scratch on limited data may suffer from overfitting, particularly when the dataset is relatively small. They may not fully exploit the rich spatial relationships and fine-grained details inherent in plant leaf images.

To address these limitations, this paper proposes a novel ensemble model called “Plant-CNN-ViT”, which combines the strengths of Vision Transformer, ResNet-50, DenseNet-201, and Xception. Each model brings unique strengths that contribute to improving the plant leaf classification accuracy. Vision Transformer (ViT) models excel at capturing global contextual information from images, allowing for effective representation learning. They can capture long-range dependencies and relationships between different parts of the leaves. ResNet-50 is known for its deep architecture, enabling the extraction of rich and multi-scale features from plant leaf images. The deep layers and residual connections of ResNet-50 facilitate the learning of complex patterns and hierarchies. DenseNet-201 enhances feature propagation through dense connections between layers. These connections promote better gradient flow and improved representation learning. DenseNet-201 can capture fine-grained details and complex variations in plant leaves. Xception models leverage depth-wise separable convolutions, reducing the computational complexity while capturing fine-grained details effectively. This makes Xception suitable for plant leaf classification tasks, where capturing intricate leaf structures and details is crucial.

The effectiveness of the proposed Plant-CNN-ViT ensemble model was assessed through rigorous evaluation on four widely used datasets: the Flavia dataset, the Folio Leaf dataset, the Swedish Leaf dataset, and the MalayaKew Leaf dataset. These datasets were specifically chosen to encompass a wide range of plant species, ensuring the evaluation of the model’s performance across various leaf characteristics and variations. Of particular note is the challenging nature of the Flavia dataset, Folio Leaf dataset, and Swedish Leaf dataset due to their limited training samples. These datasets exhibit a scarcity of training samples per class, with an approximate range of 14 to 45 samples per class. This scarcity poses significant challenges for accurate classification, as the model must learn from a small number of examples and generalize well to unseen samples. The inclusion of these datasets in the evaluation provides a comprehensive assessment of the Plant-CNN-ViT ensemble model’s ability to handle limited training samples and effectively classify plant leaves under such challenging conditions. By demonstrating robust performance on datasets with scarce training samples, the proposed model showcases its potential for practical applications in real-world scenarios where limited training data availability is a common challenge.

The main contributions of this work can be summarized as follows:

- Introduction of the Plant-CNN-ViT ensemble model, which integrates the strengths of Vision Transformer (ViT), ResNet-50, DenseNet-201, and Xception, to advance plant leaf classification. This ensemble model is designed to leverage the unique capabilities of each constituent model, enabling enhanced feature extraction and representation learning. By combining ViT, ResNet-50, DenseNet-201, and Xception, the ensemble model effectively captures the complex spatial relationships and fine-grained details present in plant leaves, leading to a notable improvement in overall classification performance.
- The efficacy of the proposed ensemble model was evaluated on widely used datasets, providing empirical evidence of its effectiveness across diverse plant species. The evaluation aimed to assess the model’s ability to handle data scarcity challenges, which are prevalent in certain datasets. Specifically, the model was subjected to the Flavia dataset, Folio Leaf dataset, Swedish Leaf dataset, and MalayaKew Leaf dataset. These datasets, with limited training samples per class, ranging from approximately 14 to 41, offer a realistic representation of the challenges encountered in plant leaf classification tasks.

## 2. Related Works

This section presents an overview of the existing research conducted in the domain of plant classification. The body of literature in this field can be broadly classified into two categories: traditional machine learning methods and deep learning approaches.

### 2.1. Traditional Machine Learning

Traditional machine learning methods in plant classification involve a two-step process. Initially, researchers perform manual feature engineering, where domain-specific features are handcrafted based on botanical knowledge and expertise. Subsequently, these engineered features are fed into machine learning algorithms for classification. Although these methods have been widely employed and have yielded notable results, their efficacy is limited by the reliance on manual feature engineering.

In an early work, Arafat et al. (2016) [1] presented a comparison of three well-known techniques for leaf classification. The authors compared the abilities of these techniques to differentiate among plant species. The evaluated techniques included the Histogram of Oriented Gradient (HOG), Color Scale Invariant Feature Transform (C-SIFT), and Maximally Stable Extremal Region (MSER). HOG, C-SIFT, and MSER features were extracted for each leaf, and classifiers were built using the SVM algorithm for HOG and the KD-Tree algorithm for C-SIFT and MSER. These techniques were evaluated on two leaf datasets: a personally built dataset and the Flavia dataset. The experimental results revealed that HOG achieved accuracy of 98% on the personal dataset and 97% on the Flavia dataset. Additionally, C-SIFT demonstrated accuracy of 98% for both datasets, while MSER achieved accuracy of 96% and 90% for the personal and Flavia dataset, respectively.

In another study, Saleem et al. (2019) [2] presented a plant classification method comprising preprocessing and segmentation, feature extraction, dimensionality reduction, and classification. The leaf was first separated from the background in the preprocessing step using segmentation, followed by the application of a Laplacian operator to smooth leaf edges and regulate small glitches. From the leaf images, shape, texture, and color features were extracted to differentiate between various types of leaves based on their geometry and texture. The feature set comprised 11 shape features, 7 statistical features, 5 venation features, and Fourier descriptors. To reduce the dimensionality of the feature vector, principal component analysis (PCA) was chosen to eliminate redundancy in the feature set dimension. A feature space of ten new features was formed by selecting only the first ten principal components, corresponding to almost 95% of the existing feature space. In the classification stage, the resultant feature vectors were classified into their respective plant species. A variety of classification methods were utilized, including k-nearest neighbor (KNN), decision tree, naïve Bayesian, and multi-support vector machine (SVM) classifiers. The proposed method achieved the highest accuracy of 98.93% on the Flavia dataset and 97.75% on the self-collected dataset when KNN was used as the classifier, without dimensionality reduction.

Trukoglu and Hanbay (2019) [3] proposed novel methods inspired by the Local Binary Patterns (LBP) method for leaf recognition. Prior to the application of the methods, morphological operations were performed for size reduction and color channel separation. The proposed approaches included filtering operations based on the regional and overall mean, using the R and G color channels in the Overall Mean-LBP (OM-LBP) and Region Mean-LBP (RM-LBP) methods, respectively. The authors also introduced a third method, ROM-LBP, which combined the parameters from OM-LBP and RM-LBP using both color channels. The attribute parameters obtained from these methods were classified using the Extreme Learning Machine method. The performance of the methods was evaluated on four datasets, where ROM-LBP achieved the highest accuracy of 98.94% on Flavia, 83.71% on ICL, and 92.92% on Foliage. Meanwhile, OM-LBP recorded the highest accuracy of 99.46% on the Swedish dataset.

In Mostajer Kheirkhah, and Asghari (2019) [4], the researchers conducted plant leaf classification with a pipeline that involved resizing images and reducing boundary artifacts using the pad-array technique. They applied a DFT filter for whitening and utilized local contrast normalization to create a pre-filter image. Subsequently, GIST features were extracted by convolving the pre-filter image with a Gabor filter bank and dividing it into four regions. Thereafter, PCA was used for feature selection and dimensionality reduction. Classification was performed using KNN, SVM, and a neural network (Patternnet). The proposed GIST features with PCA and KNN obtained the highest accuracy of 98.7% on the Flavia dataset.

Kaur and Kaur (2019) [5] used texture and color feature extraction in plant species classification. The texture features were extracted using statistical methods, specifically second-order statistics using a gray-level co-occurrence matrix (GLCM). The GLCM captured the spatial relationships between pixels and derived features such as contrast, correlation, energy, entropy, and homogeneity. The color features were extracted from segmented images using first-order statistics, including the mean, standard deviation, skewness, and kurtosis. For classification, a supervised approach using Multiclass-SVM was employed. The proposed method attained an average accuracy score of 93.26% on the Swedish Leaf Dataset.

LBP was also employed in Keivani et al. (2020) [6] as part of a hybrid approach to identify plant leaf images. In their study, the authors used a combination of GIST and LBP features, three types of geometric features, color moments, vein features, and texture features based on lacunarity for the analysis. The features were normalized after the processing phase, and a novel method called Pbest-guide binary particle swarm optimization (PBPSO) was used to reduce their number. Classification was performed using several machine learning classifiers, including KNN, decision tree, naïve Bayes, and multi-SVM. The proposed hybrid approach with decision tree achieved the highest accuracy, with 98.58% on the Flavia dataset and 90.02% on the Folio dataset.

Rajesh and Dudi (2021) [7] explored the application of five commonly used machine learning classifiers, random forest, naive Bayes, SVM, KNN, and logistic regression, for leaf image classification. The study focused on utilizing a comprehensive set of leaf features, encompassing the area, perimeter, physical length, physical width, aspect ratio, rectangularity, circularity, mean, contrast, correlation, and entropy. To assess the performance of these classifiers, the authors conducted experiments on three well-known datasets: Flavia, Swedish, and Folio. Notably, random forest demonstrated the highest accuracy of 84.11% on the Flavia dataset, 84.61% on the Swedish Leaf dataset, and 84.04% on the Folio Leaf dataset.

### 2.2. Deep Learning

In recent years, deep learning methods have gained significant attention in plant classification research. These approaches leverage the power of deep neural networks to automatically extract features directly from raw plant leaf images. Deep learning models, such as convolutional neural networks (CNNs), are designed to learn hierarchical representations and capture complex patterns in the input data. By eliminating the need for manual feature engineering, deep learning methods offer the potential to overcome the limitations of traditional approaches and achieve more accurate and robust plant classification.

In a previous study by Lee et al. (2015) [8], feature extraction was conducted using a fine-tuned convolutional neural network (CNN) on the MalayaKew Leaf dataset. The final fully connected layer was modified, replacing the original 1000 neurons with 44 neurons. The CNN architecture consisted of multiple convolutional layers with varying kernel sizes and strides, along with fully connected layers containing 4096 neurons. Two datasets, D1 and D2, were utilized for comparison. D1 encompassed complete leaf images with foreground pixels extracted through the HSV color space, while D2 consisted of cropped leaf image patches excluding the shape. The extracted features were classified using multilayer perceptron (MLP) or support vector machine (SVM). The CNN model trained with D2 and MLP as the classifier achieved the highest classification accuracy of 99.5%.

In Liu et al. (2018) [9], a plant leaf classification method was presented that was based on a ten-layer CNN. The CNN architecture used in this method was based on the LeNet model. The method utilized the feature extraction and classification capabilities of the CNN to achieve accurate leaf classification. Data augmentation techniques, including horizontal flip, vertical flip, noise, color jittering, and rotation, were applied to expand the size of the leaf database. The experimental results showed that the proposed method achieved high overall accuracy of 87.92% on the Flavia dataset.

Tan et al. (2018) [10] proposed a new CNN-based method called D-Leaf. The D-Leaf model consisted of six layers, including three convolution layers, three fully connected layers, and a softmax classification layer. The leaf images were pre-processed, and features were extracted using three different CNN models: pre-trained AlexNet, fine-tuned AlexNet, and D-Leaf. These features were then classified using five machine learning techniques: SVM, ANN, k-NN, NB, and CNN. A conventional morphometric method based on Sobel segmented veins was used for benchmarking. The D-Leaf features with ANN achieved testing accuracy of 90.38%, 94.63%, and 98.09% on the MalayaKew dataset, Flavia dataset, and Swedish Leaf dataset, respectively.

A multiscale fusion convolutional neural network (MSF-CNN) architecture was described in Hu et al. (2018) [11]. The MSF-CNN consisted of three basic units: CBR (convolutional, batch normalization, and ReLU layers), max pooling, and average pooling units. The convolutional layers utilized 3 × 3 filters, while the max pooling layers employed 3 × 3 windows. The average pooling layer utilized a 4 × 4 window. Strides of 1 and 2 pixels were applied to the convolutional and max pooling layers, respectively. Bilinear interpolation was employed for image downsampling. The MSF-CNN architecture processed four different input image sizes (256 × 256, 128 × 128, 64 × 64, and 32 × 32) to learn discriminative features at different depths. Feature fusion between different scales was achieved through the concatenation of feature maps. This multiscale fusion mechanism progressively handled multiscale images and aggregated discriminative information in the final features. On the MalayaKew Leaf dataset (D2), the MSF-CNN achieved accuracy of 99.82%.

Kaya et al. (2019) [12] investigated five classification models for plant classification, which included an end-to-end CNN model, a cross-dataset fine-tuned CNN model, pre-trained AlexNet and VGG16 models, different combinations of pre-trained AlexNet and VGG16 as feature extractors with linear discriminant analysis (LDA) and linear kernel SVM as classifiers, and pre-trained AlexNet and VGG16 as feature extractors with a recurrent neural network (RNN) as a classifier. Among these models, the pre-trained VGG16 and LDA achieved the highest classification accuracy of 99.10% on the Flavia dataset, while both the pre-trained VGG16 and CNN-RNN obtained the highest accuracy of 99.11% on the Swedish Leaf dataset. The pre-trained AlexNet with LDA yielded the highest accuracy of 96.20% on the UCI dataset.

Anubha et al. (2019) [13] conducted a study on plant species recognition using both traditional methods and deep learning approaches. The traditional method involved extracting features such as shape features (Hu moments), texture features (Haralick texture, LBP), and color features (color channel statistics). The deep learning approach used pre-trained models, specifically VGG16, VGG19, Inception-v3, and Inception-ResNet-v2, as feature extractors. The extracted features were then classified using several machine learning techniques, including linear discriminant analysis, logistic regression, classification and regression trees, naïve Bayes, KNN, random forest, and bagging classifier. The results showed that VGG16 with logistic regression achieved accuracy of 97.14% for the Leaf12 dataset, while VGG19 with the logistic regression classifier resulted in accuracy of 96.53%, 96.25%, and 99.41% for the Folio, Flavia, and Swedish Leaf datasets, respectively.

Riaz et al. (2020) [14] proposed a multi-path multi-convolutional neural network (MPF-CNN) for plant species identification. The deep feature learning architecture consisted of multiple CNN blocks, max-pooling layers, a flatten layer, and a softmax layer for classification. The features from each block were concatenated, and the overall concatenation results were aggregated to obtain the final discriminative features. The MPF-CNN architecture utilized convolution filters of size 5 × 5, 3 × 3, and 1 × 1 in all blocks, along with a 3 × 3 filter size for the max-pooling layers. The MPF-CNN achieved accuracy of 98.71% on the MalayaKew dataset (D2).

Litvak et al. (2022) [15] recently contributed to the field of plant species classification by introducing the Urban Planter dataset. This dataset consists of 1500 images categorized into 15 plant species categories, and the authors evaluated various pre-trained CNN models to classify the plant species. These models included VGG16, VGG19, Xception, Inception-ResNet-v2, Inception-v3, DenseNet-201, and MobileNet-v2. In addition, the authors investigated the effect of different pre-training approaches on the models using the ImageNet and Oxford102 datasets. The experimental results demonstrated that the DenseNet-201 model pre-trained on the ImageNet dataset achieved the highest accuracy of 96% on the Urban Planter dataset.

Arun and Viknesh (2022) [16] explored several pre-trained models, namely AlexNet, EfficientNet B0 to B7, ResNet50, and Xception. These models were trained on a plant leaf image dataset that consisted of leaf images from eleven unique plant species [18]. The experimental results revealed that EfficientNet-B5 performed better in classifying leaf images compared to the other pre-trained models, with the highest accuracy of 99.75%.

Beikmohammadi et al. (2022) [17] employed three CNN models for the purpose of plant classification. The first model, referred to as S-LeafNET, was designed with five CBR layers, four max pooling layers, and an average pooling layer. S-LeafNET was utilized to analyze the margins and overall shapes of binary leaf segments. The second model, W-LeafNET, consisted of seven CBR layers accompanied by seven max pooling layers. W-LeafNET was employed to analyze the colors, shapes, and venations of the complete leaf images. As for the third model, P-LeafNET, it involved fine-tuning a MobileNet architecture and was primarily used to classify leaf patches. The final predictions were obtained by combining the outputs of all three models through a voting mechanism, resulting in the creation of SWP-LeafNET. Remarkably, SWP-LeafNET achieved accuracy of 99.67% on the Flavia dataset and 99.81% on the MalayaKew dataset.

The existing work in plant classification has explored different approaches and techniques to achieve promising classification results. Studies have utilized various methods, such as CNN models, pre-trained models (e.g., VGG16, AlexNet, DenseNet-201), feature extraction techniques (e.g., shape, texture, color), dimensionality reduction (e.g., PCA), and different classifiers (e.g., KNN, decision tree, SVM) for plant species recognition. Despite the significant progress made in plant classification, there exist research gaps that can be addressed to further improve the accuracy. One limitation is the focus on using individual models or approaches, neglecting the potential benefits of combining multiple models or architectures. By leveraging the strengths of different models, it is possible to enhance the classification accuracy. Additionally, while the Vision Transformer architecture has shown outstanding performance in image classification tasks, its potential in plant classification remains unexplored.

To address these research gaps, this paper proposes an ensemble method that combines the Vision Transformer, ResNet-50, DenseNet-201, and Xception architectures for plant species classification. The ensemble aims to leverage the unique characteristics of each architecture to achieve improved accuracy and robustness. The inclusion of the Vision Transformer architecture introduces the attention mechanism, which can capture global dependencies and relationships within plant images. This allows the model to focus on important regions and patterns, enhancing its understanding of the image content. The ResNet-50, DenseNet-201, and Xception architectures, known for their strong feature extraction capabilities, complement the Vision Transformer’s attention-based features. They can effectively capture low-level and high-level visual features, including intricate structures and textures within plant images. By combining these four architectures in an ensemble, the model benefits from their synergistic strengths, potentially leading to enhanced classification performance.

## 3. Plant-CNN-ViT

**Figure 1:**
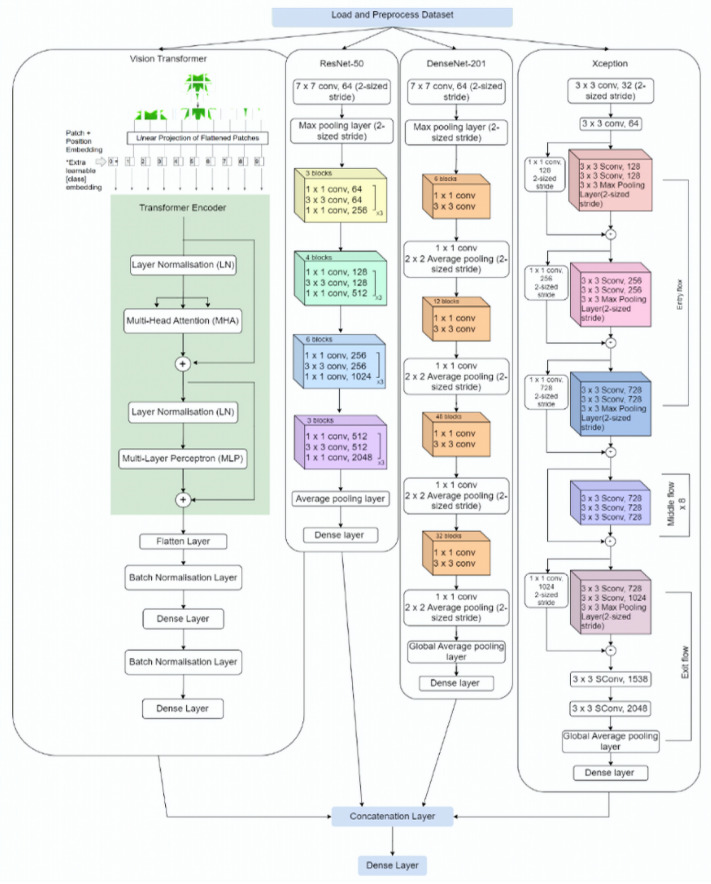
Architecture of Plant-CNN-ViT.

This paper introduces an ensemble model named “Plant-CNN-ViT” for plant leaf classification. The proposed model combines the capabilities of four pre-trained models, namely Vision Transformer, ResNet-50, DenseNet-201, and Xception. The architecture of the Plant-CNN-ViT model is depicted in Figure 1, illustrating the integration of these models for plant leaf classification. The plant leaf images from the datasets undergo individual processing in each model. The activation maps obtained from the final dense layer of each model are then concatenated and forwarded to the classification layer. This approach enables the comprehensive utilization of the extracted features from each model, facilitating the more robust classification of plant leaves.

### 3.1. Vision Transformer

Transformers have achieved remarkable success in natural language processing tasks, largely attributed to their attention mechanisms. Building upon this concept, the Vision Transformer (ViT) [19] has emerged as a powerful architecture for image classification. The ViT architecture comprises three fundamental components.

- Patch Embedding

The plant image $x\in R^{H\times W\times C}$ is partitioned into fixed-size patches to be transformed into a sequential representation of flattened 2D patches $x_{p}\in R^{N\times \left(P^{2}\cdot C\right)}$, where *H* represents the image height, *W* denotes the image width, *C* is the number of channels, and (P,P) represents the resolution of each image patch. The number of patches *N* can be calculated as

(1) $$N=\frac{H\times W}{P^{2}}$$

Before feeding the sequence of patches into the Transformer, a linear projection is applied to the patches. During this linear projection, the patches are mapped to a *D*-dimensional vector space by multiplying them with an embedding matrix E. The output of this linear projection is referred to as a patch embedding. To enable the model to capture positional information within the image, positional embeddings $E_{pos}$ are appended to the patch embeddings. Additionally, the embedded image patches are concatenated with a learnable class token $x_{\mathrm{class}}$, which is essential for the classification process. The initial patch embedding $z_{0}$, consisting of the embedded sequence of image patches along with the class token, is computed as follows:

(2) $$z_{0}=\left[x_{\mathrm{class}};x_{p}^{1}E;x_{p}^{2}E;\cdots ;x_{p}^{N}E\right]+E_{pos},\;E\in R^{\left(P^{2}\cdot C\right)\times D},E_{pos}\in R^{(N+1)\times D}$$

Here, $x_{p}^{n}$ represents the *n*-th image patch, where n∈1,2,…,N. The resulting embedded image patches are then passed to the Transformer encoder.

- Transformer Encoder

The Transformer encoder is composed of *L* identical encoder blocks, each containing two sub-layers: a multi-head self-attention (MSA) layer and a fully connected feed-forward multi-layer perceptron (MLP) layer. In each encoder block, the *ℓ*-th layer receives the input sequence from the previous layer $z_{\ell -1}$. The input $z_{\ell -1}$ undergoes layer normalization, which normalizes the input values across the feature dimension, improving the training time and performance. Next, the output of the layer normalization is passed to the MSA layer.

The output of the MSA layer is then layer-normalized again. Finally, the output from the layer normalization is fed into the MLP layer. Residual connections, also known as skip connections, are employed in the encoder block to facilitate the flow of information between non-adjacent layers. These connections allow gradients to propagate through the network without being affected by non-linear activation functions, addressing the issue of vanishing gradients. The gradient flow in the *ℓ*-th encoder layer is defined as

(3) $$z_{\ell }^{\prime }=\mathrm{MSA}\left(\mathrm{LN}\left(z_{\ell -1}\right)\right)+z_{\ell -1},\;\;\ell =1,\ldots ,L$$

(4) $$z_{\ell }=\mathrm{MLP}\left(\mathrm{LN}\left(z_{\ell }^{\prime }\right)\right)+z_{\ell }^{\prime },\;\;\ell =1,\ldots ,L$$

where LN denotes layer normalization.

The MSA consists of a linear layer, self-attention layer, concatenation layer, and a final linear layer. In the MSA, multiple self-attention operations are performed in parallel based on the number of heads *k*. In each head, the *D*-dimensional patch embedding z is multiplied by three weight matrices $U_{q}$, $U_{k}$, and $U_{v}$ to obtain the query (q), key (k), and value (v) matrices. The multiplication operation in each head is defined as

(5) $$\left[q,k,v\right]=\left[zU_{q},zU_{k},zU_{v}\right],\;\;U_{q},U_{k},U_{v}\in R^{D\times D_{h}}$$

The obtained matrices q, k, and v are then projected into *k* subspaces, and the weighted sum over all values V is calculated. Attention weights are computed in each head based on the relationship between each pair of elements (i,j), using the dot product of $q^{i}$ and $k^{j}$. The resulting dot product indicates the importance of patches in the sequence. The dot product of q and k is computed, and a softmax function is applied to obtain the weights on the values, as follows:

(6) $$A=\mathrm{softmax}\left(\frac{qk^{⊺}}{\sqrt{D_{h}}}\right),\;\;A\in R^{N\times N}$$

where $D_{h}=\frac{D}{k}$.

The self-attention matrices are then concatenated and passed through a single linear layer with a learnable weight matrix $U_{msa}$, resulting in

(7) $$\mathrm{MSA}\left(z\right)=\left[{\mathrm{SA}}_{1}\left(z\right);{\mathrm{SA}}_{2}\left(z\right);\ldots ;{\mathrm{SA}}_{k}\left(z\right)\right]U_{msa},\;\;U_{msa}\in R^{k\cdot D_{h}\times D}$$

Each head of the MSA captures information from different aspects and positions, allowing the model to encode intricate features in parallel.

- Classification

The classification of the ViT model is performed by the multi-layer perceptron (MLP), which consists of two fully connected layers with the Gaussian error linear unit (GeLU) activation function. The GeLU activation function applies a weight to the inputs based on their values rather than their signs. Unlike the ReLU function, GeLU can produce both positive and negative outputs, and it exhibits a higher degree of curvature. This property allows the GeLU function to better approximate complex functions compared to the ReLU function.

In the encoder, the last layer selects the first token of the sequence, $z_{L}^{0}$, and generates the image representation r by applying layer normalization. The resulting r is then passed through a small MLP head, which consists of a single hidden layer with the sigmoid function, for classification purposes. The image representation of the sequence is obtained as follows:

(8) $$r=\mathrm{LN}\left(z_{L}^{0}\right)$$

### 3.2. ResNet-50

ResNet-50 [20] introduced the concept of residual connections to address the degradation problem in deep neural networks. The ResNet-50 architecture consists of 50 layers, including convolutional layers, pooling layers, fully connected layers, and shortcut connections. The key innovation of ResNet-50 is the incorporation of residual blocks, which allow the network to learn residual functions rather than directly fitting the desired underlying mapping.

Each residual block in ResNet-50 contains two or three convolutional layers with batch normalization and ReLU activation functions. These layers are followed by a skip connection that directly connects the input of the residual block to its output. This skip connection enables the gradient to flow more easily during training, mitigating the problem of vanishing gradients.

The skip connection can be mathematically represented as follows:

(9) $$y=F(x,W_{i})+x$$

where x represents the input to the residual block, $F(x,W_{i})$ denotes the residual function implemented by the convolutional layers with weights $W_{i}$, and y represents the output of the residual block.

By introducing these residual connections, ResNet-50 effectively tackles the problem of information degradation in deep networks, allowing the model to be deeper while maintaining or improving its performance. The skip connections enable the gradient to propagate through the network more effectively, enabling the learning of deeper and more complex features.

### 3.3. DenseNet-201

DenseNet-201 [21] is characterized by its densely connected layers and efficient information flow. The DenseNet-201 architecture is composed of multiple dense blocks, each consisting of several densely connected convolutional layers. In contrast to traditional CNN architectures, where information flows sequentially through the layers, DenseNet-201 introduces dense connections that allow for direct connections between layers at different depths. This dense connectivity promotes feature reuse and facilitates the efficient flow of information throughout the network.

Each dense block in DenseNet-201 is composed of multiple densely connected layers, typically consisting of a combination of convolutional layers, batch normalization, and activation functions. The output of each layer is concatenated with the feature maps from all preceding layers within the same dense block. This concatenation operation can be mathematically represented as

(10) $$x_{l}=\left[h_{0},h_{1},\ldots ,h_{l-1}\right]$$

where $x_{l}$ represents the output of the *l*-th layer within the dense block, and $h_{i}$ denotes the feature maps from the *i*-th preceding layer.

By densely connecting the layers within each dense block, DenseNet-201 encourages feature reuse, allowing the network to access a rich set of features at each layer. This leads to a more compact model and alleviates the vanishing gradient problem, enabling the efficient training of deep networks. Furthermore, DenseNet-201 incorporates transition layers between the dense blocks to control the number of feature maps and reduce the spatial dimensions. These transition layers typically consist of a convolutional layer followed by a pooling layer, which helps to compress the information and improve the computational efficiency.

### 3.4. Xception

Xception [22] is known for its innovative approach to the design of convolutional layers, which aims to capture more fine-grained spatial information and enhance feature representation.

The Xception architecture is based on the concept of depth-wise separable convolutions, which decouple the spatial and channel-wise dimensions of the convolution operation. Unlike traditional convolutional layers that perform convolutions across both spatial and channel dimensions simultaneously, Xception introduces separate operations for each dimension, leading to improved efficiency and effectiveness.

The key idea behind depth-wise separable convolutions is to apply spatial convolutions independently for each channel and then combine the results through pointwise convolutions. Mathematically, the depth-wise separable convolution operation can be represented as

(11) $$Y=DW\left(X\right)*PW\left(Y^{\prime }\right)$$

where X represents the input feature maps, DW denotes the depth-wise convolution operation, PW represents the pointwise convolution operation, $Y^{\prime }$ denotes the intermediate feature maps, and Y represents the output feature maps.

By separating the spatial and channel-wise operations, Xception significantly reduces the number of parameters and computations required compared to traditional convolutional layers. This enables deeper network architectures with fewer parameters, facilitating better learning and improved representation capability. In addition to depth-wise separable convolutions, Xception also incorporates residual connections, inspired by the ResNet architecture. These connections allow the network to learn residual functions and mitigate the vanishing gradient problem, enabling the effective training of very deep networks. Furthermore, Xception employs a modular design with repeated sequences of convolutional blocks followed by pooling layers. This design facilitates the extraction of hierarchical features at different levels of abstraction, enabling the network to capture both low-level and high-level visual patterns.

### 3.5. Fusion of Model Output

After training the models, the next step in the classification process involves combining the outputs of multiple models. This can be achieved by using a concatenation layer, which merges the outputs of the individual models into a single tensor. The concatenation layer concatenates the individual output tensors along a specified axis, resulting in a merged activation map from all the models. This concatenated representation is then passed through the rectified linear unit (ReLU) activation function. The ReLU activation function introduces non-linearity to the output of each unit, allowing the network to learn complex patterns and improve the expressive power of the model. Finally, the softmax activation function is used in the classification layer of Plant-CNN-ViT to obtain normalized probabilities for each class.

## 4. Experiments and Discussion

In this section, the datasets used for the performance evaluation are described, along with the comparison results with existing methods.

### 4.1. Datasets

The proposed Plant-CNN-ViT is evaluated using four publicly available datasets: the Flavia dataset, Folio Leaf dataset, Swedish Leaf dataset, and MalayaKew Leaf dataset. Table 1 provides a summary of the plant leaf datasets.

**Figure 2:**
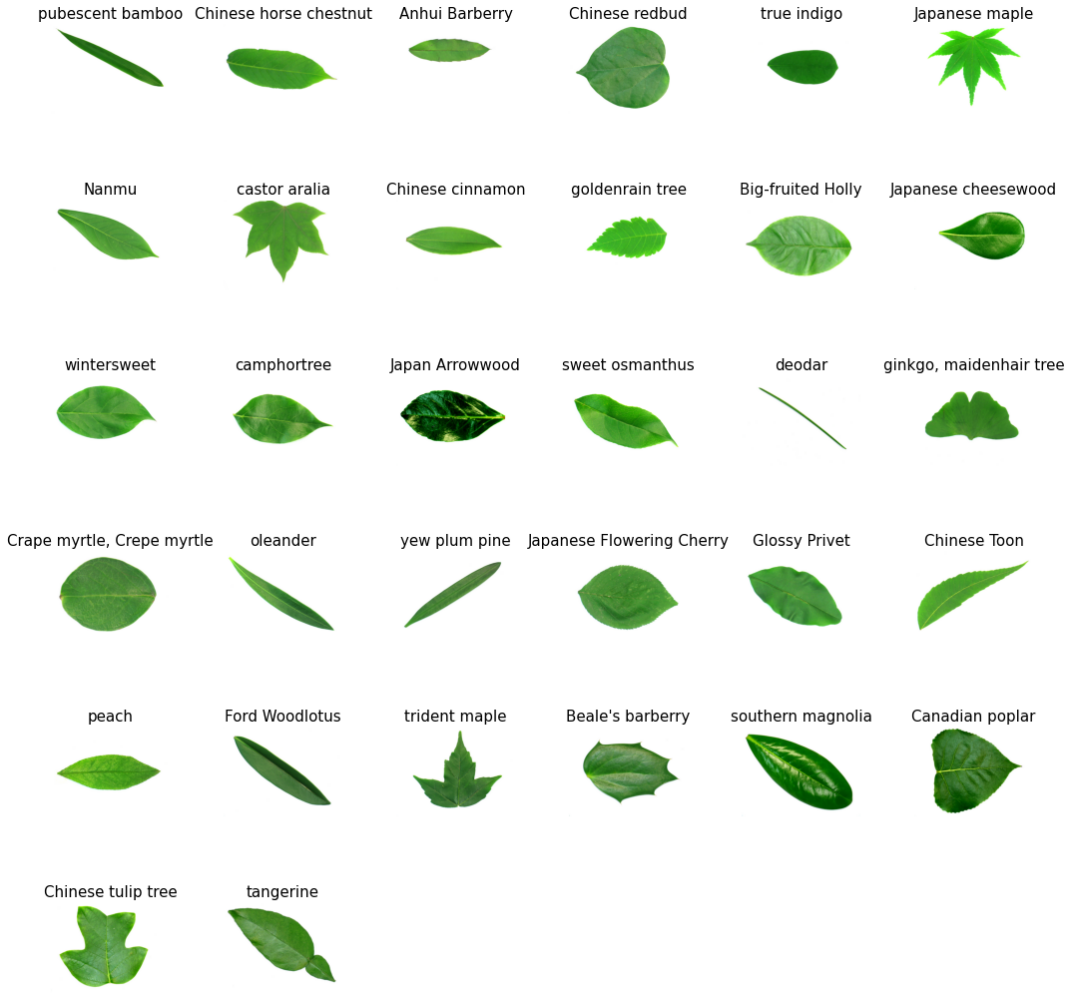
Sample images of the Flavia dataset.

**Figure 3:**
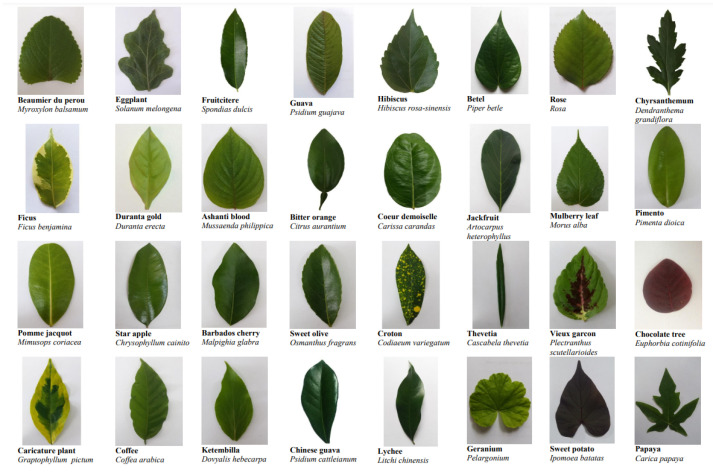
Sample images of the Folio Leaf dataset [24].

**Figure 4:**
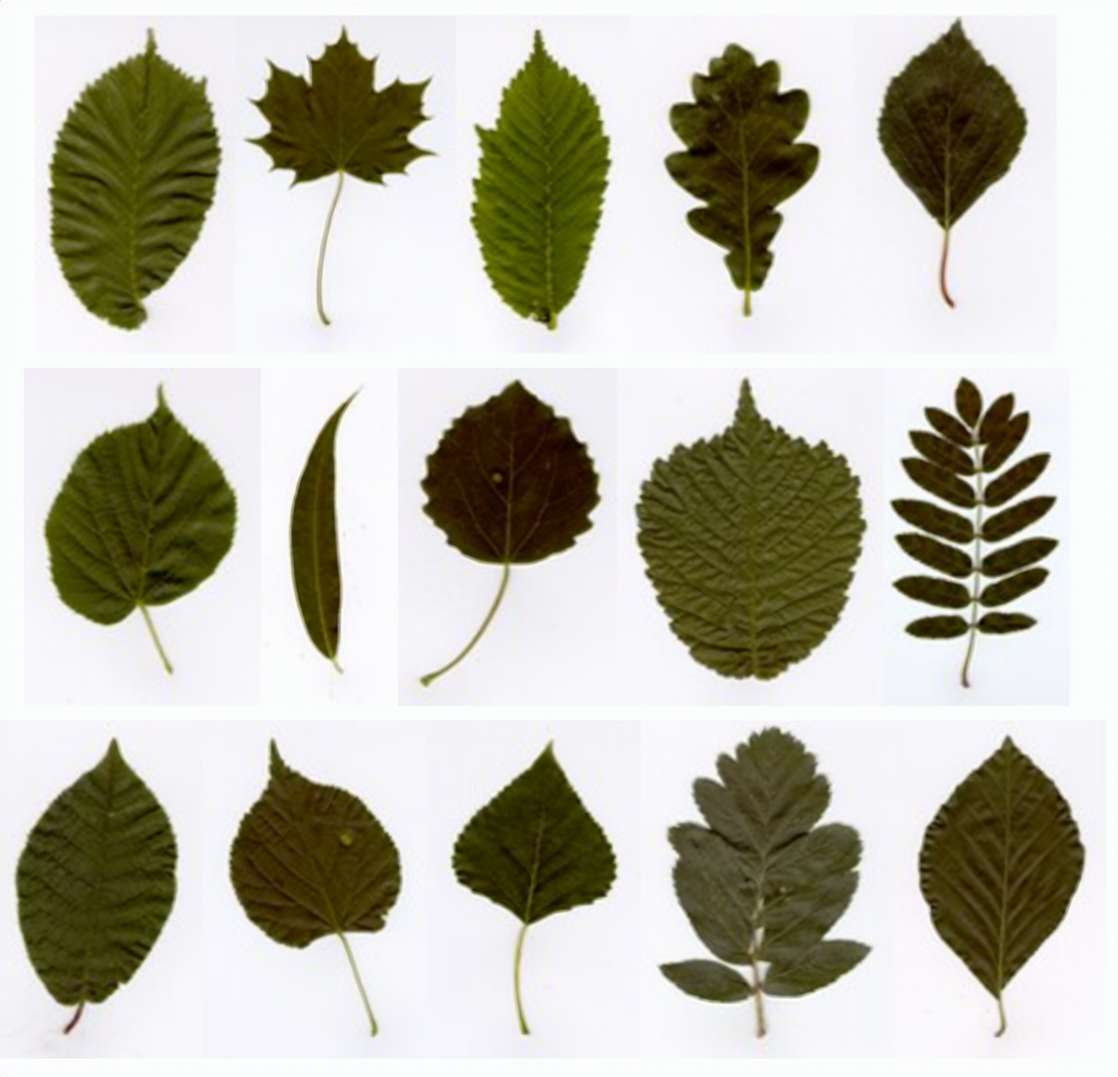
Sample images of the Swedish Leaf dataset [25].

**Figure 5:**
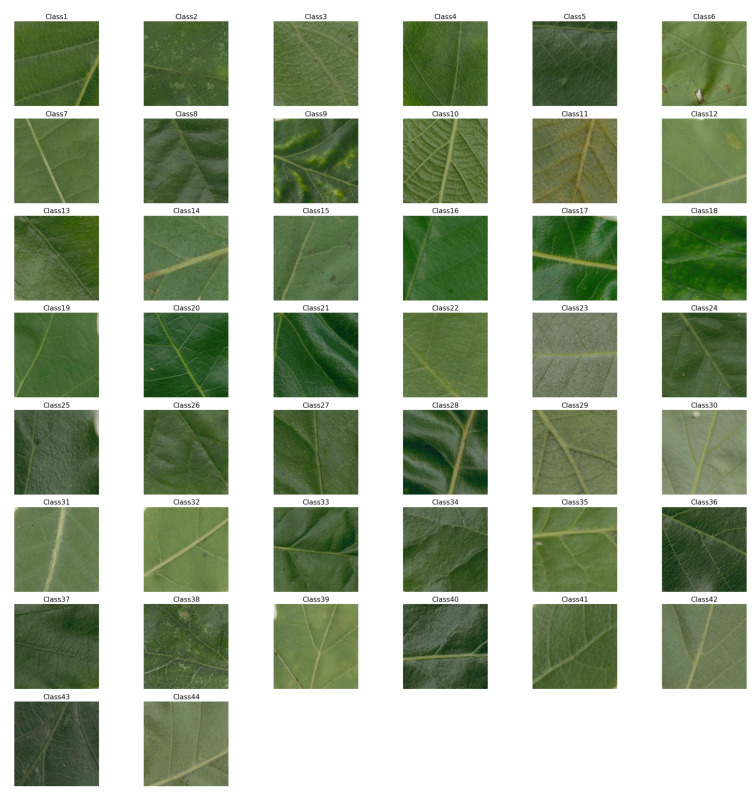
Sample images of the MalayaKew Leaf dataset (D2).

- **Flavia Dataset:** The Flavia dataset, created by Wu et al. [23], comprises a total of 32 classes and 1907 images. Each class contains 50 to 77 images. The dataset is divided into three subsets: training, validation, and testing. The training set consists of 1323 images (70%), the validation set contains 178 images (10%), and the testing set consists of 406 images (20%). Figure 2 presents sample images from the Flavia dataset.
- **Folio Leaf Dataset:** The Folio Leaf dataset, developed by Munisami et al. [24], is the smallest dataset in this experiment, consisting of 637 images. It encompasses 32 classes, with each class containing 20 images, except for two classes. The dataset was captured in the University of Mauritius’s farm and nearby locations. The training set comprises 445 images (70%), the validation set contains 62 images (10%), and the testing set consists of 130 images (20%). Some sample images from the Folio Leaf dataset are illustrated in Figure 3.
- **Swedish Leaf Dataset:** The Swedish Leaf dataset, created by Oskar J. O. Söderkvist [25], includes 15 species of Swedish trees. Each class consists of 75 images, resulting in a total of 1125 images in this dataset. The dataset is divided into three sets, with 675 images in the training set (60%), 105 images in the validation set (10%), and 345 images in the testing set (30%). Figure 4 shows sample images from the Swedish Leaf dataset.
- **MalayaKew Leaf Dataset:** The MalayaKew Leaf dataset, introduced by Lee et al. (2015) [8], was collected from the Royal Botanical Gardens, Kew, England. It comprises two variations, namely D1 and D2, where D1 represents whole leaf images and D2 contains patches extracted from the leaves. For this experiment, D2 is employed, which includes 43,472 patches from 44 different classes. The training dataset consists of 34,672 images, while the testing dataset consists of 8800 images. The training dataset is further divided into two parts, with 80% (27,720 images) allocated to training and 20% (6952 images) for the validation set. Figure 5 showcases sample images from the MalayaKew Leaf dataset (D2).

### 4.2. Comparison with Existing Methods

This section compares the performance of the proposed Plant-CNN-ViT with the existing works in plant leaf classification. The experimental setup of Plant-CNN-ViT involved several important variables and settings. The image size used for the Flavia, Folio Leaf, and Swedish Leaf datasets was set to 224 × 224 pixels, while, for the MalayaKew Leaf dataset, a smaller image size of 96 × 96 pixels was used. The batch size, which determines the number of samples processed in each iteration, was set to 32. The learning rate of the pre-trained models was set to 0.0001. During the training process, the pre-processed images were individually fed into the four pre-trained models for feature extraction and training. The outputs from these models were then combined and inputted into a concatenation layer. Following this, two fully connected layers were added to the model. The first dense layer consisted of 50 units and employed the rectified linear unit (ReLU) activation function, while the unit size of the second dense layer corresponded to the number of classes in the dataset. The activation function used for the second dense layer was the softmax function.

Table 2 presents a comparison of various methods for plant leaf classification on the Flavia dataset. Among the existing methods, machine learning techniques such as KNN, ROM-LBP, and PBPSO with decision tree or SVM showed better performance, surpassing 90% accuracy. However, their performance was still exceeded by the proposed Plant-CNN-ViT. Notably, deep-learning-based approaches demonstrated notable advancements. Models such as VGG16, VGG19, and CNN-RNN achieved high accuracy in the range of 99%, demonstrating the potential of convolutional neural networks. The SWP-LeafNet model further improved the performance, reaching an impressive accuracy score of 99.67%.

**Figure 6:**
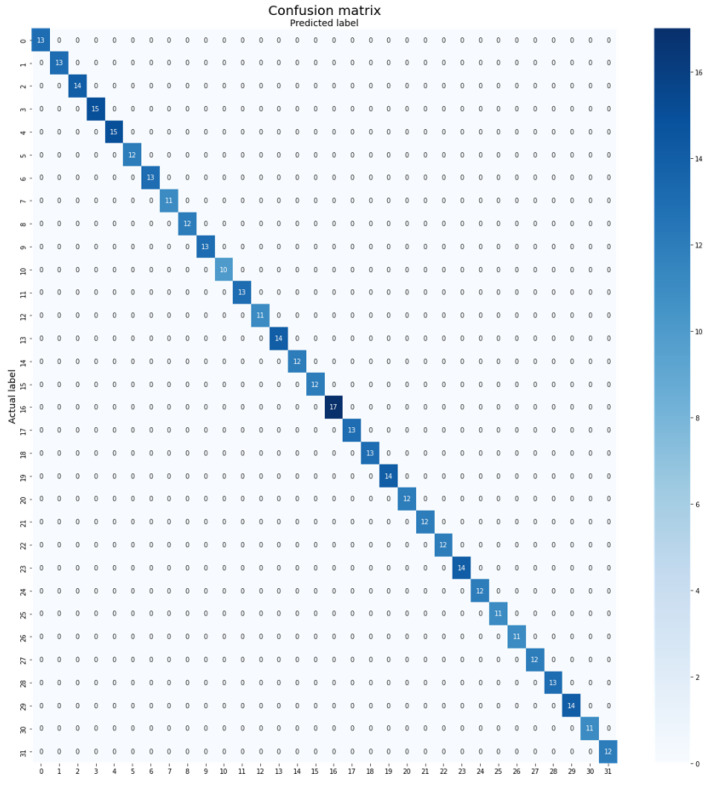
Confusion matrix of Plant-CNN-ViT on the Flavia dataset.

Despite the limited availability of training samples in the Flavia dataset, with only an average of 41 samples per class, the proposed Plant-CNN-ViT model demonstrated exceptional performance by achieving 100% classification accuracy. This outstanding outcome can be attributed to the adoption of an ensemble approach that combines multiple powerful architectures, harnessing their complementary features and collectively enhancing the classification performance. The integration of Vision Transformer, ResNet-50, DenseNet-201, and Xception models synergistically contributes to the model’s capability in capturing intricate leaf characteristics and accomplishing highly accurate plant leaf classification. The confusion matrix depicted in Figure 6 provides a visual representation of the classification performance of the Plant-CNN-ViT model on the Flavia dataset.

Table 3 presents a comparison of different methods for plant leaf classification on the Folio Leaf dataset, which is characterized by an average of 14 training samples per class. Consequently, the achieved accuracy of all methods was relatively lower. Traditional machine learning techniques, including PBPSO with decision tree, SVM, naive Bayes, and KNN, demonstrated moderate accuracy ranging from 81.30% to 90.02%. The random forest and logistic regression methods achieved accuracy of 84.04% and 77.65%, respectively. Encouragingly, deep-learning-based approaches yielded promising results. Notably, the combination of VGG19 with logistic regression achieved accuracy of 96.53%, showcasing the effectiveness of deep convolutional neural networks in the context of leaf classification tasks.

**Figure 7:**
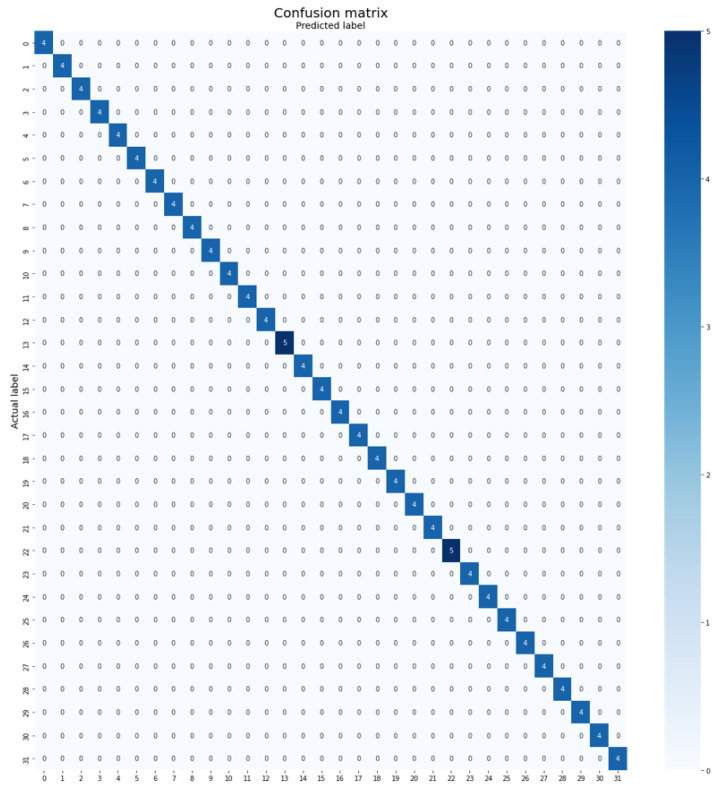
Confusion matrix of Plant-CNN-ViT on the Folio Leaf dataset.

The proposed Plant-CNN-ViT, which combines the Vision Transformer, ResNet-50, DenseNet-201, and Xception models, achieved remarkable accuracy of 100%. This exceptional performance is attributed to the ensemble approach, which effectively harnesses the diverse strengths of these architectures. The Vision Transformer model captures global patterns and long-range dependencies, while the ResNet-50, DenseNet-201, and Xception models excel in capturing intricate local features. By integrating these models, the Plant-CNN-ViT ensemble demonstrates a comprehensive understanding of leaf characteristics, resulting in highly accurate classification even with limited dataset availability. The confusion matrix of Plant-CNN-ViT on the Folio Leaf dataset can be observed in Figure 7.

Table 4 provides a comparison of the different methods for plant leaf classification on the Swedish Leaf dataset. Despite the Swedish Leaf dataset being considerably small, with an average of 45 training samples per class but only 15 classes, notable accuracy was achieved by the different approaches. Among the existing methods, OM-LBP achieved notable accuracy of 99.46% by effectively capturing local leaf texture information. Another method, GLCM with Multiclass-SVM, achieved respectable accuracy of 93.26% by leveraging gray-level co-occurrence matrix features and support vector machines. The D-Leaf with ANN approach achieved accuracy of 98.09%, highlighting the potential of deep-learning-based methods.

**Figure 8:**
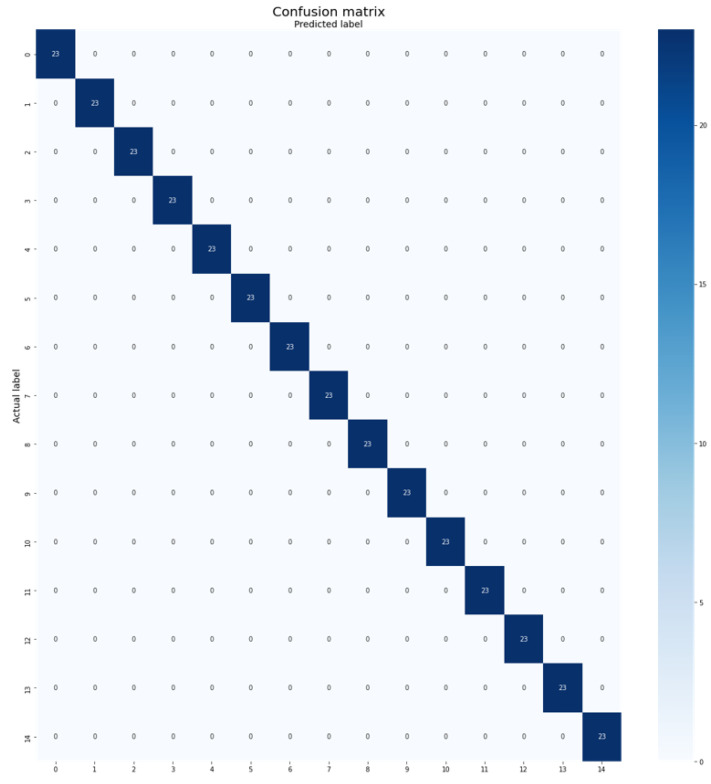
Confusion matrix of Plant-CNN-ViT on the Swedish Leaf dataset.

However, the proposed Plant-CNN-ViT method stood out by achieving impressive accuracy of 100%, surpassing all other approaches. This superior performance can be attributed to several factors. Firstly, the ensemble strategy allows the model to combine the strengths of multiple architectures, enabling it to capture diverse and complementary information from the leaf data. The Vision Transformer, ResNet-50, DenseNet-201, and Xception models excel in different aspects of feature extraction and representation learning, leading to a comprehensive understanding of leaf characteristics. Figure 8 provides the confusion matrix obtained by Plant-CNN-ViT when evaluated on the Swedish Leaf dataset.

Table 5 provides a summary of the performance of various methods for plant leaf classification on the MalayaKew Leaf dataset (D2), which is the largest dataset, with an average of over 600 training samples per class and a high number of classes. Comparatively, MLP and SVM achieved high accuracy of 99.50% and 99.30%, respectively, utilizing traditional machine learning algorithms. The D-Leaf with ANN approach achieved accuracy of 90.38% by employing an artificial neural network for leaf representation learning. The MSF-CNN method achieved impressive accuracy of 99.82% by utilizing a multiscale fusion convolutional neural network architecture, which captures discriminative information through feature fusion. Similarly, the MPF-CNN approach achieved accuracy of 98.71% by optimizing the network parameters and enhancing the feature learning through a multi-path multi-convolutional neural network. Another method, SWP-LeafNet, achieved accuracy of 99.81% by combining the predictions of multiple deep neural networks.

**Figure 9:**
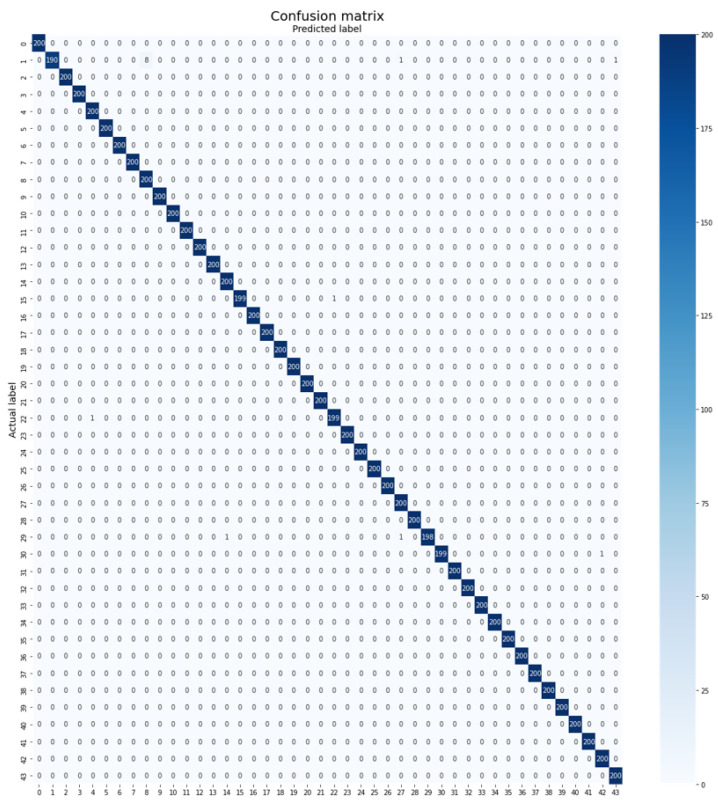
Confusion matrix of Plant-CNN-ViT on the MalayaKew Leaf dataset (D2).

The challenging nature of the MalayaKew Leaf dataset (D2) is highlighted by the presence of patches with high similarity, posing additional difficulties for classification. However, the proposed Plant-CNN-ViT method outperformed all other methods by achieving accuracy of 99.83%. The deep learning architectures utilized in the Plant-CNN-ViT model excel in learning hierarchical representations from complex data. This enables the model to extract intricate leaf features at multiple levels of abstraction, leading to improved classification accuracy. The inclusion of the Vision Transformer introduces attention mechanisms that enhance the model’s ability to focus on relevant leaf patterns and capture fine-grained details. Figure 9 visualizes the confusion matrix of the MalayaKew Leaf dataset obtained using the Plant-CNN-ViT model.

## 5. Conclusions

This paper introduces a novel approach to plant leaf classification, termed Plant-CNN-ViT, which combines the strengths of ViT, ResNet-50, DenseNet-201, and Xception models. The proposed method leverages the feature maps extracted from each of these models and concatenates them using a dedicated concatenation layer, followed by a final classification layer.

The ViT model excels in capturing long-range dependencies in images through self-attention mechanisms, enabling it to learn global contextual information. ResNet-50, known for its residual connections, enables effective feature extraction by mitigating the vanishing gradient problem. DenseNet-201 facilitates feature reuse and gradient flow through dense connections, leading to enhanced representation learning. Xception, characterized by its depth-wise separable convolutions, achieves a good balance between model complexity and computational efficiency. The performance of the proposed Plant-CNN-ViT model on different leaf datasets is impressive. It achieves accuracy scores of 100.00%, 100.00%, 100.00%, and 99.83% on the Flavia, Swedish, Folio, and MalayaKew datasets, respectively. This performance demonstrates the effectiveness and robustness of the Plant-CNN-ViT model in plant leaf classification tasks across various datasets.

One potential limitation of the proposed method is the integration of multiple models, which introduces increased computational complexity to the classification pipeline. However, considering the achieved accuracy and the relatively low urgency of plant leaf classification, this limitation can be deemed tolerable. In addition, the proposed Plant-CNN-ViT model can be extended to tackle other related tasks in plant biology, such as plant disease detection, by leveraging the learned representations and incorporating additional domain-specific data.

## Figures and Tables

**Table 1:** Summary of plant leaf datasets.

Dataset	Number of Classes	Total Number of Images	Training	Validation	Testing
Flavia Dataset	32	1907	1323 (70%)	178 (10%)	406 (20%)
Folio Leaf Dataset	32	637	445 (70%)	62 (10%)	130 (20%)
Swedish Leaf Dataset	15	1125	675 (60%)	105 (10%)	345 (30%)
MalayaKew Leaf Dataset	44	43,472	27,620 (80%)	6952 (20%)	8800

**Table 2:** Comparison of results on Flavia dataset.

Methods	Accuracy (%)
HOG with SVM [1]	97.00
C-SIFT with KD tree [1]	98.00
MSER with KD tree [1]	90.00
KNN [2]	98.93
ROM-LBP [3]	98.94
Cosine KNN [4]	95.50
SVM [4]	89.90
Patternnet Neural Network [4]	72.20
PBPSO with Decision Tree [6]	98.58
PBPSO with SVM [6]	96.12
PBPSO with Naive Bayes [6]	92.01
PBPSO with KNN [6]	94.89
Random Forest [7]	84.11
SVM [7]	79.05
Logistic Regression [7]	84.11
KNN [7]	80.10
Naive Bayes [7]	72.25
CNN [9]	87.92
D-Leaf with ANN [10]	94.63
VGG16 and LDA [12]	99.10
VGG16 [12]	99.11
CNN-RNN [12]	99.11
VGG19 with Logistic Regression [13]	96.25
SWP-LeafNet [17]	99.67
ViT	99.75
ResNet-50	98.28
DenseNet-201	99.51
Xception	97.04
Plant-CNN-ViT (proposed)	100.00

**Table 3:** Comparison of results on Folio Leaf dataset.

Methods	Accuracy (%)
PBPSO with Decision Tree [6]	90.02
PBPSO with SVM [6]	88.02
PBPSO with Naive Bayes [6]	81.30
PBPSO with KNN [6]	89.21
Random Forest [7]	84.04
SVM [7]	60.63
Logistic Regression [7]	77.65
KNN [7]	70.21
Naive Bayes [7]	72.34
VGG19 with Logistic Regression [13]	96.53
ViT	96.92
ResNet-50	96.15
DenseNet-201	95.38
Xception	97.69
Plant-CNN-ViT (proposed)	100.00

**Table 4:** Comparison of results on Swedish Leaf dataset.

Methods	Accuracy (%)
OM-LBP [3]	99.46
GLCM with Multiclass-SVM [5]	93.26
Random Forest [7]	84.61
SVM [7]	79.28
Logistic Regression [7]	84.02
KNN [7]	76.03
Naive Bayes [7]	73.07
D-Leaf with ANN [10]	98.09
VGG19 with Logistic Regression [13]	99.41
ViT	98.26
ResNet-50	98.26
DenseNet-201	98.55
Xception	95.07
Plant-CNN-ViT (proposed)	100.00

**Table 5:** Comparison of results on MalayaKew Leaf dataset.

Methods	Accuracy (%)
MLP [8]	99.50
SVM (Linear) [8]	99.30
D-Leaf with ANN [10]	90.38
MSF-CNN [11]	99.82
MPF-CNN [14]	98.71
SWP-LeafNet [17]	99.81
ViT	97.85
ResNet-50	95.67
DenseNet-201	91.98
Xception	90.28
Plant-CNN-ViT (proposed)	99.83

## Data Availability

Not applicable.
